# A Spermidine Derivative Ameliorates Dextran Sulfate Sodium-Induced Colitis in Mice by Inhibiting the MAPK4/AKT Signaling Pathway

**DOI:** 10.3390/foods14071110

**Published:** 2025-03-23

**Authors:** Yuxin Zhang, Zeyuan Deng, Hongyan Li, Zeyin Jiang

**Affiliations:** 1 State Key Laboratory of Food Science and Resources, Nanchang University, Nanchang 330047, China; zyx05101999@163.com (Y.Z.); dengzy@ncu.edu.cn (Z.D.); lihongyan@ncu.edu.cn (H.L.); 2 International Institute of Food Innovation, Nanchang University, Nanchang 330051, China

**Keywords:** spermidine derivative, ulcerative colitis, intestinal mucosal barrier, MAPK4/AKT

## Abstract

Ulcerative colitis (UC) is a chronic inflammatory bowel disease characterized by recurrent episodes and an inability to achieve a complete cure. The spermidine derivative (di-*p*-coumaroyl-caffeoyl spermidine, SPDD), as a key alkaloid, exhibits unique health benefits. However, it has not yet been reported whether SPDD can improve dextran sulfate sodium (DSS)-induced colitis in mice. Herein, we investigated the effects and mechanisms of SPDD on DSS-induced colitis in mice. SPDD was successfully purified from rose bee pollen and was found to have a protective effect on colitis, evidenced by reduced disease activity index (DAI) scores and colonic inflammation, increased colonic length and upregulated the expression of tight junction proteins (TJs) in the model (*p* < 0.05). Importantly, the IL-17 signaling pathway showed significant enrichment by RNA sequencing (RNA-seq) technology with SPDD treatment, which resulted in the downregulation of MAPK4 expression (*p* < 0.05). Furthermore, SPDD weakened the interaction between MAPK4 and AKT, resulting in a decrease in the phosphorylation level of AKT, thereby reducing the expression of IL-6, IL-1β, iNOS, and COX-2, and alleviating colitis (*p* < 0.05). In addition, SPDD treatment also ameliorated TNF-α-induced inflammation in Caco-2 cells. Overall, our study demonstrated that SPDD reversed colonic inflammation in colitis mice through the MAPK4/AKT pathway and might be a promising candidate for UC intervention.

## 1. Introduction

Inflammatory bowel disease (IBD), encompassing ulcerative colitis (UC) and Crohn’s disease (CD), is a chronic condition characterized by immune system dysfunction. Although the mortality rate is not high, it significantly impacts patients’ quality of life [1]. UC is marked by cyclical flare-ups and difficulty in maintaining remission. In 2023, the prevalence of ulcerative colitis was estimated to be 5 million cases around the world, and the incidence is increasing worldwide [2,3].

The pathogenesis of UC may involve genetic predisposition, environmental influences, impaired intestinal barrier function, aberrant immune responses, and dysbiosis of the gut microbiota [4]. Common symptoms experienced by patients include fatigue, fever, nausea, diarrhea, hematochezia, and arthritis [5,6,7,8], with severe complications such as toxic megacolon in extreme cases [9,10]. Treatment for UC is typically divided into two phases: induction of remission and maintenance therapy. During the induction phase, medications commonly used include 5-aminosalicylic acid (5-ASA) and corticosteroids. Conventional therapies (e.g., amino salicylates, corticosteroids, and immunosuppressants), as well as biologicals (e.g., anti-tumor necrosis factor (TNF) and anti-integrin antibodies), are available to treat UC [11,12]. Despite these therapeutic approaches, some patients struggle to achieve long-term remission and may require surgical intervention, which can be associated with complications and long-term risks [13,14].

The exploration of personalized treatment strategies for UC is currently a focal point of research. Natural products have garnered attention due to their potential anti-inflammatory properties. For instance, studies have indicated that citrus peel polyphenols can alleviate symptoms of acute colitis in mice [15]. Additionally, it has been demonstrated that ginsenoside Rg1 can reduce the inflammatory response in DSS-induced colitis in mice [16]. These findings reveal the potential of bioactive compounds from natural sources in the treatment of UC, offering a new perspective for the development of safer and more effective therapeutic strategies.

Spermidine is a low molecular weight natural polyamine with an aliphatic triamine structure featuring two nitrogen atoms connected by three or four methylene groups. It is widely distributed across various organisms [17,18]. Spermidine derivatives are conjugates of hydroxycinnamic acids and amines, inheriting the properties of their parent compounds. Due to the potential combinations of multiple parent compounds, these derivatives exhibit diverse metabolic, chemical, and biological activities [19].

Phenylethanoids are either a class of phenolic compounds or a class of spermidine derivatives, widely present in plant reproductive organs and seeds, playing a crucial role in plant growth, development, and responses to environmental stress as metabolic intermediates or end products in various biological processes [20]. Previous studies indicate that phenylethanoids are abundant in the fruit and peel of *Lycium barbarum* (goji berry) and possess a multitude of bioactivities, including anti-aging, blood pressure-lowering, blood glucose-lowering, and immunomodulatory effects [21,22,23]. These conjugated phenylethanoids not only retain the properties of their parent phenolic acids and amines but also exhibit unique health benefits. Nakamura et al. isolated N1, N5, N10-tri-*p*-coumaroyl spermidine from Artemisia annua and demonstrated its inhibitory effect on HIV-1 protease activity, an activity not observed with its parent compounds, spermidine and caffeic acid [24]. Further research by Nakase et al. elucidated that feruloyl spermidine induced the contraction of bladder strips and inhibited norepinephrine-induced contraction of urethral strips [20]. Moreover, studies have indicated that phenylethanoids possess favorable inhibitory effects on inflammation [25].

In this study, we successfully isolated and purified an SPDD from rose bee pollen and comprehensively evaluated its therapeutic effects in a DSS-induced mouse model of UC, hoping to provide new experimental evidence and a theoretical basis for UC treatment with nature phytochemicals.

## 2. Materials and Methods

### 2.1. Chemicals and Reagents

SPDD was extracted from rose bee pollen (Shanxi, China). DSS (MW, 36,000–50,000 Da) was purchased from Aladdin Biotechnology Co., Ltd. (Shanghai, China). The following primary antibodies were used: MAPK4 (PU179773), AKT (T55561), anti-phospho-Akt (Ser473) (T40067), anti-phospho-Akt (Ser308) (T40068), which were purchased from Abmart Biotechnology Co., Ltd. (Shanghai, China), and β-Tubulin (sc-8035) (Santa Cruz Biotechnology, Santa Cruz, CA, USA). All other reagents used were of analytical grade.

### 2.2. Extraction, Purification, and Identification of SPDD

SPDD Extraction: Rose bee pollen was mixed with 80% methanol (1:30, *w*/*v*) and vortexed. The mixture was subjected to ultrasonic extraction for 20 min, followed by centrifugation at 4000 rpm for 10 min. The supernatant was collected, and the residue was re-extracted three times under the same conditions. The combined supernatants were concentrated under reduced pressure at 40 °C to obtain the crude plant extract. The crude plant extract was then dissolved in methanol for further purification.

SPDD Purification and Identification: SPDD was separated and purified using preparative high-performance liquid chromatography (preparative HPLC) with a Sepax GP-C18 column (21.2 mm × 250 mm, 5 μm). The mobile phase consisted of water (A) and acetonitrile (B), with the following gradient elution program: 0–15 min, 20–45% B; 15–25 min, 45% B; 25–30 min, 45–46% B. The flow rate was maintained at 10 mL/min, and detection was performed at a wavelength of 280 nm. After purification, the samples were immediately stored in a −80 °C freezer to prevent degradation or contamination. Prior to analysis, the samples were thawed at 4 °C and filtered through a 0.22 μm membrane filter to remove particulate matter. Specifically, preparative HPLC was employed for the separation of the compound, and LC-Q-TOF MS was used for structural identification. The chemical structure was determined by comparing the fragment ions. The detailed experimental procedures and parameter settings were referenced from the study by Yuan et al. [26].

### 2.3. Animals and Experimental Design

Male C57BL/6J mice (7–8 weeks of age) were purchased from Sipeifu Biotechnology Co., Ltd. (Beijing, China) and housed in a controlled environment with regulated temperature (22 ± 2 °C) and humidity (50 ± 10%), under a 12-h light/dark cycle. The mice were allowed a one-week acclimatization period during which they had free access to standard laboratory chow and drinking water. After the acclimatization period, the mice were randomly assigned to different groups based on their body weights to ensure uniform baseline conditions at the start of the experiment. All experimental procedures were conducted in accordance with the guidelines of the Ethics Committee of Nanchang University and were approved (Approval No.: SYXK (Jiangxi) 2021-0004).

To evaluate the therapeutic effects of SPDD on colitis, the mice were divided into three groups (*n* = 8 per group): (1) Control group: mice were given normal drinking water and administered 200 μL of 0.5% sodium carboxymethyl cellulose (CMC-Na) via gavage; (2) Model group: mice were given 3% DSS (MW 36,000–50,000) in their drinking water and administered 200 μL of 0.5% CMC-Na via gavage; (3) SPDD group: mice were given 3% DSS in their drinking water and administered 200 μL of SPDD (dissolved in 0.5% CMC-Na) (20 mg/kg BW) [27]. The experimental duration was 7 days. Following the acclimatization period, mice in the Model and SPDD groups were given a 3% DSS solution in their drinking water. Concurrently, mice in the SPDD group were administered SPDD (dissolved in 0.5% CMC-Na) via gavage at the same time each day for 7 days. In addition, to ensure that the mice consumed the required amount of DSS, the DSS solution was replaced with a fresh one at fixed times daily, and the water intake of each cage of mice was recorded. By calculating the average daily water intake per mouse, the DSS dosage intake was ensured to be in accordance with the experimental design. Meanwhile, the body weight and health status of the mice were regularly monitored to assess the effects of DSS intake.

### 2.4. DAI Scores

During the experiment, the disease activity index (DAI) was recorded daily, encompassing weight loss, stool characteristics, and fecal blood. The scoring criteria were as follows: Weight loss (0: <1%, 1: 1–5%, 2: 5–10%, 3: 10–20%, 4: >20%); stool consistency (0: normal, 2: loose stools, 4: diarrhea); and fecal blood (0: normal, 2: occult blood, 4: gross bleeding). The DAI score for each animal was the sum of these three scores. At the end of the experiment, all mice were humanely euthanized, and colon tissues were collected and fixed in 4% paraformaldehyde for histopathological analysis [28].

### 2.5. H and E Staining and Histological Analysis

Colon tissues fixed in 4% paraformaldehyde for over 48 h were embedded in paraffin and sectioned into approximately 5 μm slices. Hematoxylin and eosin (H and E) staining was then performed. The stained sections were observed under an upright optical microscope and imaged accordingly (Nikon Eclipse E100, Tokyo, Japan). Histological damage was scored based on three criteria as described by Wlodarska: crypt damage (0: normal, 3: severe crypt damage), inflammation (0: normal, 3: severe inflammation), and muscle thickening (0: no thickening, 3: marked thickening). The histological damage score for each animal was the sum of these three scores [29].

### 2.6. Immunohistochemistry Analysis

Paraffin-embedded colon tissue sections were dewaxed with water, followed by antigen retrieval, serum blocking (30 min at room temperature), endogenous peroxidase blocking, primary antibody incubation (overnight at 4 °C), secondary antibody incubation (50 min at room temperature), DAB staining, dehydration, and mounting with neutral gum for detection. Ultimately, the divided portions were examined using a fluorescence microscope (Nikon E100, Tokyo, Japan), and the fluorescence strength was measured using ImageJ software (National Institutes of Health, Bethesda, MD, USA) [30,31].

### 2.7. RNA-Seq Analysis

The RNA-seq analysis was conducted by Novogene Technology Co., Ltd. (Tianjin, China). Total RNA was extracted from the colon tissues of four randomly selected mice from the Model and SPDD groups. The quantity and purity of RNA were determined using the NanoDrop NC-2000 (Thermo Scientific, Waltham, MA, USA). PolyA-enriched mRNA was isolated from the total RNA and fragmented into approximately 300 bp segments. Double-stranded cDNA was synthesized using these fragments as templates to construct the sequencing libraries. The libraries were sequenced on the Illumina platform using next-generation sequencing technology with paired-end sequencing. Differential gene expression (DEGs) analysis was performed using the DESeq software (version 1.18.0). Genes with a *p*-value of <0.05 and a log2|fold change| > 1 were considered significantly differentially expressed between the two groups. Statistical enrichment analyses of Gene Ontology (GO) and Kyoto Encyclopedia of Genes and Genomes (KEGG) pathways for the DEGs were conducted using the topGO and KAA programs, respectively.

### 2.8. Cell Experiment

Cell Culture: Caco-2 cells were cultured in Dulbecco’s Modified Eagle Medium (DMEM; Solarbio, Beijing, China) supplemented with 15% fetal bovine serum (VivaCell, Shanghai, China) and 0.1% penicillin-streptomycin solution, under a humidified atmosphere of 5% CO_2_ at 37 °C.

Caco-2 cells were seeded into 6-well plates (NEST Biotechnology, Wuxi, China). Cells in the control group were cultured in a DMEM medium for 24 h. Cells in the model group and the SPDD group were first cultured in DMEM medium for 12 h, followed by induction of cellular inflammation with 20 ng/mL TNF-α for 12 h. Subsequently, the SPDD group was treated with 1 μg/mL SPDD for 24 h, after which the subsequent indicators were measured.

### 2.9. Real-Time Quantitative PCR Analysis

Total RNA was extracted from cells or tissue samples using TRIzol reagent (Biomiga Biochemicals, San Diego, CA, USA). Upon agarose gel electrophoresis, the RNA sample exhibited distinct bands corresponding to 5S, 18S, and 28S ribosomal RNAs, indicating high-quality RNA. The concentration and purity of the RNA were further confirmed by a spectrophotometer with an A260/A280 ratio greater than 2.0. Subsequently, 1 µg of total RNA was reverse transcribed into cDNA using a reverse transcription kit from Vazyme (Nanjing, China). Quantitative real-time PCR was performed as described previously [32]. Gene expression was normalized to the expression of glyceraldehyde-3-phosphate dehydrogenase (GAPDH), and the relative change in gene expression was calculated using the 2^−ΔΔCt^ method [33]. Primer sequences are shown in Table S1.

### 2.10. Western Blot Analysis

Caco-2 cells were seeded into 6-well plates (NEST Biotechnology, Wuxi, China). After 24 h, the cells were transfected with MAPK4 plasmids, and then the cells were stimulated with SPDD (1 μg/mL) for 24 h before harvest.

The cells in each well were extracted using RIPA lysis buffer (Millipore, Billerica, MA, USA) containing protease and phosphatase inhibitors (Roche Diagnostics, Rotkreuz, Switzerland), followed by centrifugation at 12,000 rpm for 15 min at 4 °C. The supernatant was collected and mixed with 5× protein loading buffer, followed by boiling at 95 °C for 10 min. The extracted proteins were quantified using a BCA protein assay kit (Millipore, Billerica, MA, USA). The protein samples were diluted to consistent concentrations and separated by SDS-PAGE electrophoresis. After incubation with MAPK4, AKT, and anti-phospho-Akt antibodies, signals were detected using the enhanced chemiluminescence detection system (Tanon 5200, Shanghai, China), and images were captured [34]. ImageJ software was used to quantify the band intensities from the Western blot images (National Institutes of Health, Bethesda, MD, USA).

### 2.11. Co-Immunoprecipitation (Co-IP) Analysis

To explore whether SPDD affects the interaction between MAPK4 and AKT, Caco-2 cells were co-transfected with MAPK4-GFP and AKT-Flag plasmids. After 24 h, the cells were treated with TNF-α (20 ng/mL) and SPDD (1 μg/mL) for 24 h, respectively, followed by washing with PBS. After 24 h, the cells were lysed with NP40 buffer on ice for 30 min. After centrifugation, carefully collect the supernatant from each group. To each collected supernatant, add 25 μL of Anti-GFP Nanobody Agarose Beads (catalog number KTSM1301 from AlpalifeBio, Shenzhen, China) or Anti-Flag (catalog number KTSM1308 from AlpalifeBio, Shenzhen, China), respectively. Incubate the mixture at 4 °C for 5 h. Then, the supernatant was removed and the beads were added 1 mL of washing buffer (10 mM Tris-HCl pH 7.5, 150 mM NaCl, 0.5 mM EDTA). Agarose beads were collected by centrifugation. The above experiment was repeated at least three times. 50 μL of 2× loading buffer was used to re-suspend the agarose beads. The next day, the immunoprecipitates were washed five times for immunoblotting assays.

### 2.12. Statistical Analysis

Experimental data were expressed as the mean ± standard error of the mean (SEM). All datasets were subjected to normality testing using the Shapiro-Wilk test (*p* > 0.05) and homogeneity of variance testing using Levene’s test (*p* > 0.05) via SPSS 26.0 software (IBM, New York, NY, USA). Data that met these criteria were analyzed using one-way analysis of variance (ANOVA) followed by the Least Significant Difference (LSD) test. Non-parametric tests were employed for datasets that did not meet these assumptions. The level of statistical significance was set at *p* < 0.05.

## 3. Results

### 3.1. Purification and Identification of SPDD

**Figure 1:**
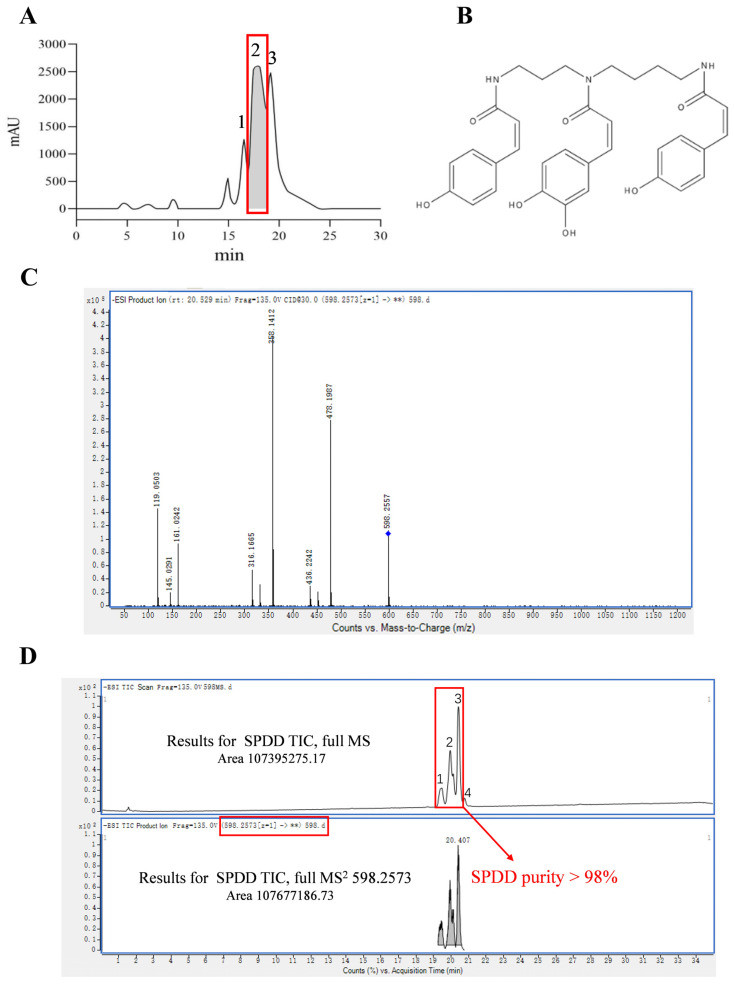
Purification and Identification of SPDD. (**A**) Preparative liquid phase separation profile. (**B**) The chemical structure of SPDD. (**C**) Mass spectra of the compound in negative ion mode (LC-QTOF-MS/MS). (**D**) Total ion chromatogram for LC-QTOF-MS/MS identification.

This study aims to extract and identify a bioactive compound from rose bee pollen. Utilizing preparative HPLC technology, the target compound was successfully isolated (Figure 1A). The identification results of peaks 1, 2, and 3 in Figure 1A are shown in Table S1. Subsequently, peak 2 was collected and further analyzed and identified via liquid chromatography-quadrupole time-of-flight mass spectrometry (LC-Q-TOF MS) [26]. In negative ion mode, the quasi-molecular ion peak [M-H]^−^ of the target compound was observed with an m/z value of 598.2557. Detailed analysis of the MS/MS data identified key fragment ions with m/z values of 119.0503, 316.1665, 358.1412, and 478.1987. These fragment ions provided crucial information regarding the structure of the compound. By comparing the fragmentation pathway with the relevant literature, a possible structure for the compound was proposed [26]. The chemical structure is shown in Figure 1B. Ultimately, the compound was identified as di-*p*-coumaroyl-caffeoyl spermidine (Figure 1C). Additionally, analysis of the peak area ratio confirmed purity of the compound exceeded 98%, further validating the effectiveness of our separation strategy (Figure 1D). The identification results of peaks 1, 2, 3, and 4 in Figure 1D are shown in Table S2.

### 3.2. SPDD Ameliorated the Weight Loss, Disease Activity Index, and Tissue Damage in Mice with Colitis

**Figure 2:**
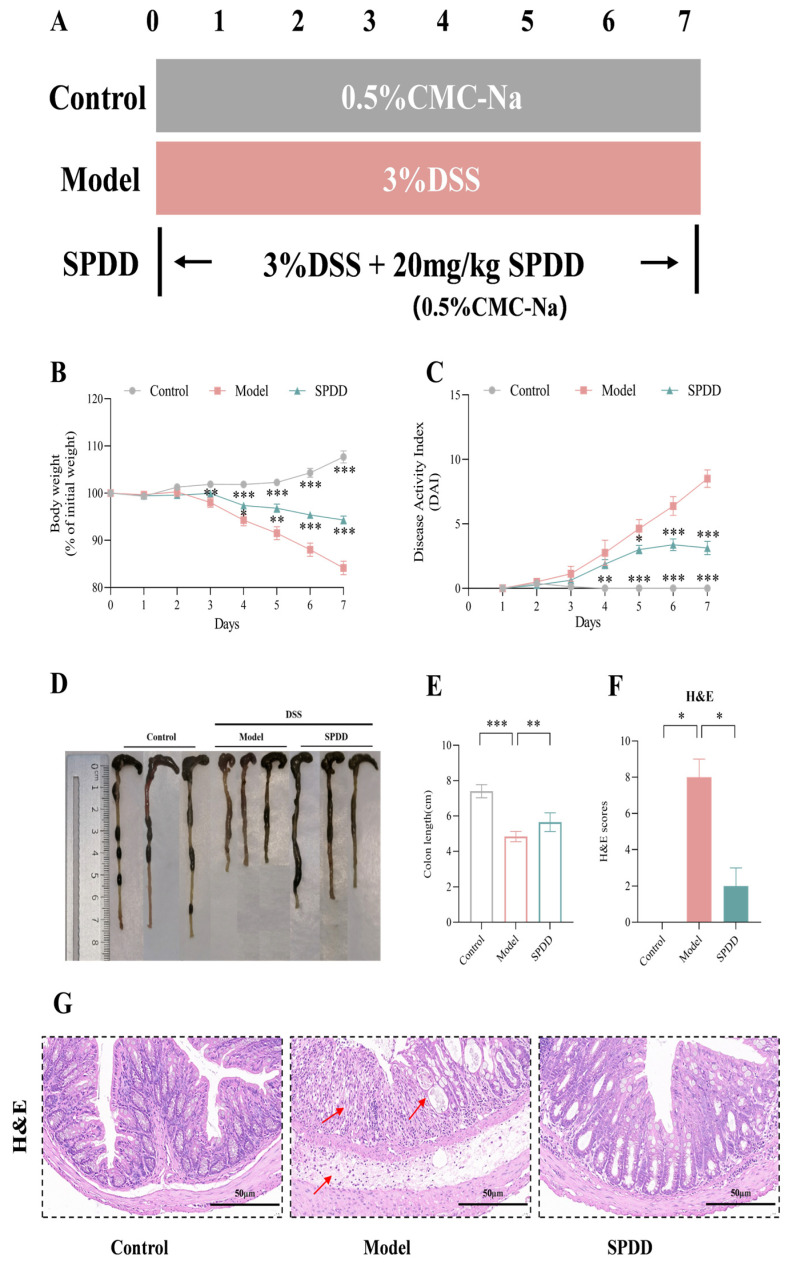
Effects of SPDD on the symptoms of mice with DSS-induced colitis. (**A**) Experimental Design. (**B**) Weight change. (**C**) DAI score. (**D**) Representative pictures of the colon. (**E**) Colon length. (**F**) Pathological score of colonic sections. (**G**) H and E-stained pictures of the colon (30×). Values are mean ± SEM (*n* = 8). * *p* < 0.05; ** *p* < 0.01 and *** *p* < 0.001 indicate comparisons between the Model group and the Control or SPDD group. The scale is marked in the (**G**) (scale bar = 50 μm). Note: The red arrows indicate the areas of damage in the colonic tissues of mice.

To evaluate the ameliorated effects of SPDD on UC, C57BL/6J mice drank ad lib water containing 3% DSS for 7 days while intragastric SPDD (Figure 2A). Body weight and DAI scores were monitored daily throughout the experiment. The results indicate that the DSS-treated model group showed a significant decrease in body weight starting from the third day (*p* < 0.01), which progressively worsened over subsequent days. Additionally, the model group developed watery stools and rectal bleeding, leading to a significantly higher DAI score compared to the control group (*p* < 0.001). However, the SPDD-treated group experienced a markedly reduced weight loss (*p* < 0.001), with a decrease in watery stools and bleeding, resulting in a significantly lower DAI score compared to the model group (*p* < 0.001) (Figure 2B,C). Furthermore, SPDD treatment significantly alleviated the DSS-induced reduction in colon length, indicating that SPDD effectively ameliorated the pathological characteristics of UC in mice (*p* < 0.01) (Figure 2D,E).

Furthermore, histological analysis and pathological scoring of colon tissues were conducted. In the DSS-induced UC model, colon tissues exhibited severe damage, including irregular or absent glandular structures, extensive ulcer formation, and significant epithelial cell loss accompanied by inflammatory cell infiltration [35]. Compared with the control group, the colonic tissue in the model group was subjected to more severe damage, with the histological score being significantly higher than that of the control group (*p* < 0.05). Compared to the model group, the SPDD-treated group showed significantly lower histopathological scores (*p* < 0.05), indicating a protective effect of SPDD on colon tissue (Figure 2F,G). The SPDD group maintained well-preserved glandular structures, orderly arranged goblet cells, only mild submucosal edema, and no significant inflammatory cell infiltration. The histological score of this group was significantly lower than that of the model group (*p* < 0.05). These findings confirm the successful establishment of the UC mouse model and demonstrate the therapeutic potential of SPDD against DSS-induced colitis.

### 3.3. SPDD Improved Intestinal Barrier Function in Mice with Colitis

**Figure 3:**
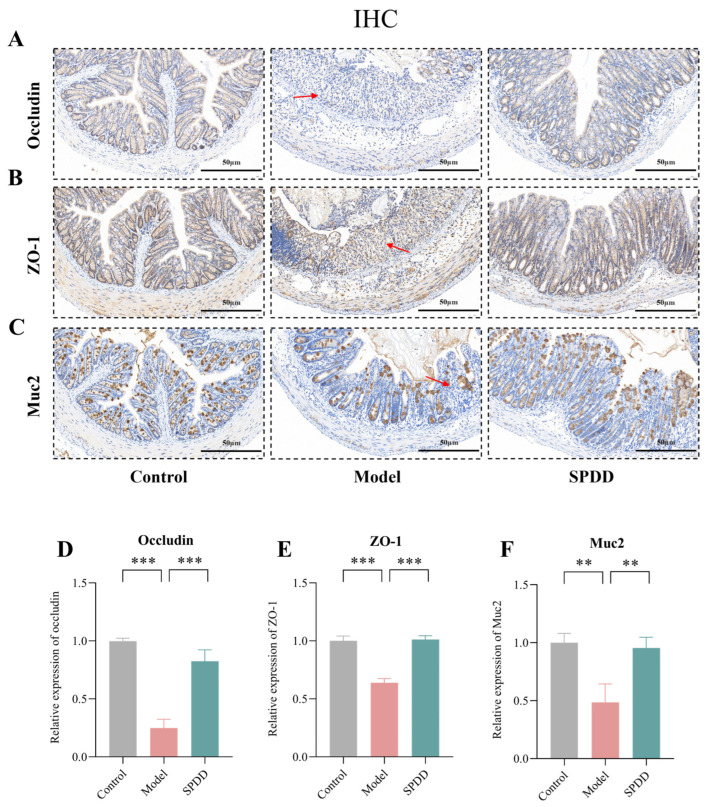
Effects of SPDD on colon pathological and colon barrier damage of mice with DSS-induced colitis. Representative images of immune-histochemical analysis of the expression of (**A**) Occludin, (**B**) ZO-1, and (**C**) Muc2 of colon section (30×). (**D**–**F**) Relative expressions of Occludin, ZO-1, and Muc2 in colon tissue. Values are mean ± SEM (*n* = 3). ** *p* < 0.01 and *** *p* < 0.001 indicate comparisons between the Model group and the Control or SPDD group. Arrows in histopathology stains represent tissue lesions. The scale is marked in (**A**–**C**) (scale bar = 50 μm). Note: The red arrows indicate areas with low expression of mucin proteins.

The integrity of the intestinal barrier is maintained by tight junction proteins such as ZO-1 and Occludin, while the mucosal barrier is regulated by colonic mucins such as Muc2 [36]. Under UC conditions, mucins and tight junction proteins are significantly downregulated, leading to increased permeability of the intestinal barrier to microbial ligands and harmful metabolites, which triggers systemic inflammation [37]. To further assess the protective effects of SPDD on intestinal barrier function, the expression levels of Occludin, ZO-1, and Muc2 were evaluated via immunohistochemistry. The IHC results showed that in the control group, the colonic epithelial cells displayed well-structured and abundant expression of Occludin, ZO-1, and Muc2 (Figure 3A–F). In contrast, the model group significantly reduced the expression of these proteins (*p* < 0.01), disrupting the colonic epithelium and mucosal barrier. Our experimental results further confirm that SPDD treatment significantly restored the expression of Occludin, ZO-1, and Muc2 (*p* < 0.01), indicating its beneficial role in enhancing the integrity of the intestinal barrier.

### 3.4. Transcriptomic Profiling and Pathway Analysis

**Figure 4:**
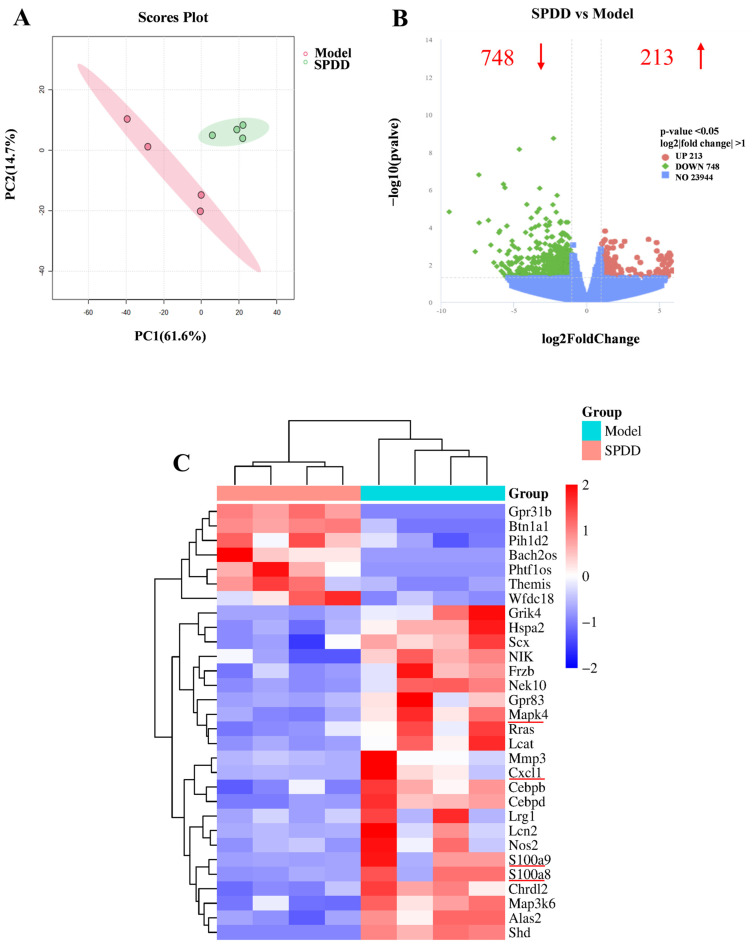

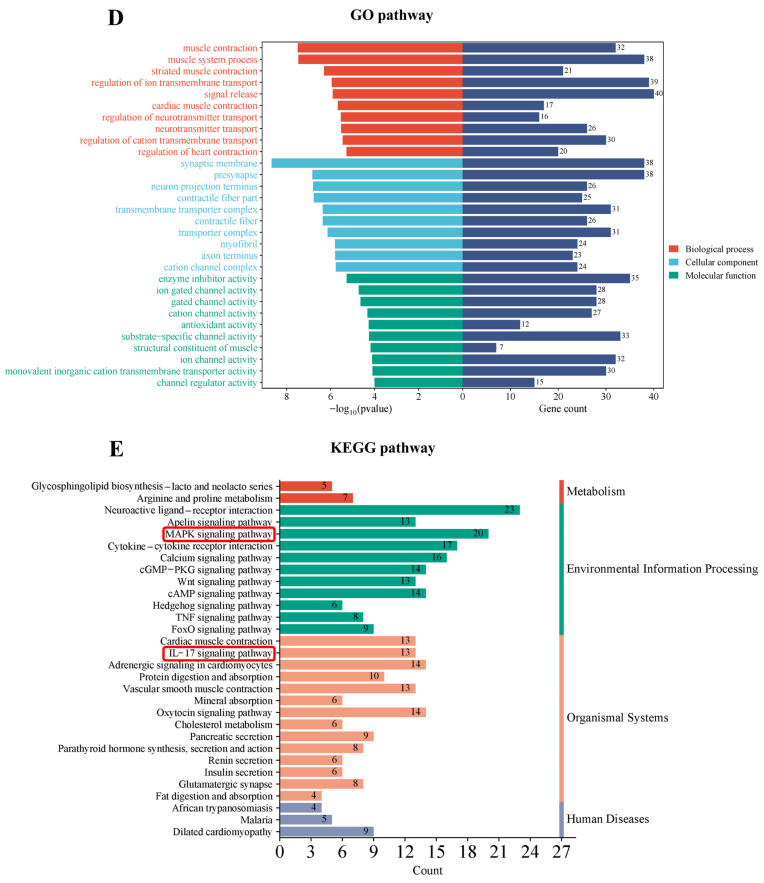

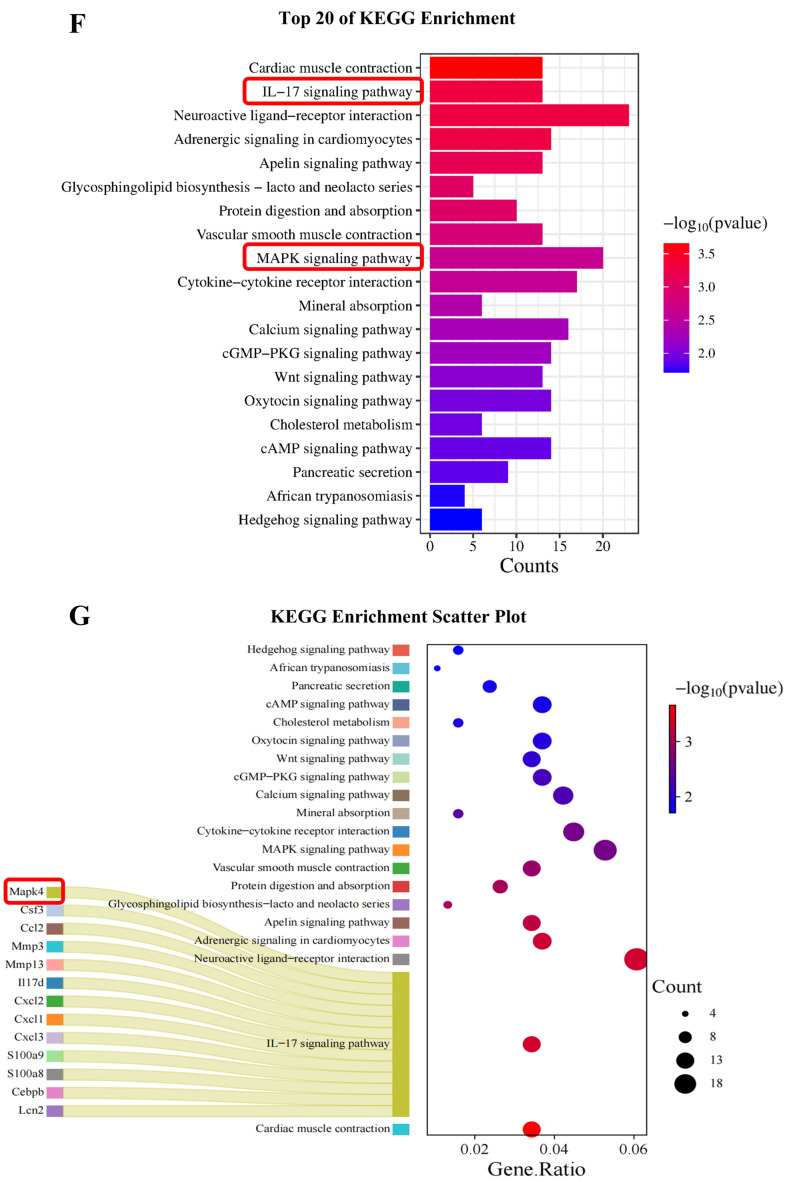
Differential gene expression between the Model and SPDD groups in the colon was investigated via RNA sequencing. (**A**) PCA score plot. (**B**) Volcano plot identifying DEGs (log2|fold change| > 1, *p*-value < 0.05), red indicates upregulated genes, green indicates downregulated genes. (**C**) Heatmap of DEGs, where red and blue indicate up-and down-regulated genes. (**D**) Differential gene GO enrichment analysis. (**E**) KEGG pathway analysis. (**F**) Top 20 enriched KEGG pathways. (**G**) KEGG Enrichment Scatter Plot.

To explore the molecular mechanisms through which SPDD ameliorates UC, transcriptome analysis (RNA-seq) was conducted to assess the mRNA levels of various genes in mouse colonic tissue. Principal component analysis (PCA) revealed that the administration of spermidine-derived compounds significantly altered the physiological state of the mice (Figure 4A). As depicted in Figure 4B, the volcano plot features the *X*-axis representing the log2 fold change, while the *Y*-axis denotes the negative logarithm (base 10) of the significance level. Differentially expressed genes (DEGs) are highlighted by red and green dots, with those on the left side indicating downregulated genes (relative to the Model group) and those on the right side representing upregulated genes (relative to the Model group). The central blue region corresponds to genes with no significant differential expression. In the comparison between the Model group and the SPDD group, a greater number of differentially expressed genes were observed. To further elucidate the extent of differential gene expression, the transcripts per million (TPM) method was employed. The criteria for selecting DEGs were set as log2|fold change| > 1 and *p*-value < 0.05. Compared with the Model group, a total of 961 DEGs were identified in the SPDD group, comprising 213 upregulated and 748 downregulated genes (Figure 4B). These DEGs were subsequently employed as the target gene set for further analysis.

A clustering analysis was performed on the 961 selected target gene sets to further elucidate the specific expression patterns of significant DEGs, with the results presented in Figure 4C. Compared with the Model group, the SPDD group exhibited significant downregulation of genes such as MAPK4, CXCL1, and S100A8/9. Previous studies have demonstrated that overexpression of MAPK4 promotes tumor progression [38]. Additionally, it has been established that Xpa-deficient mice exhibit systemic upregulation of CXCL1 following UVB exposure, which is associated with a robust inflammatory response [39]. S100A8 and S100A9 can serve as a predictive indicator for therapeutic responses in inflammation-related diseases [40]. The downregulation of these inflammation-related DEGs suggests the protective effects of SPDD against UC, thereby fundamentally elucidating the beneficial anti-inflammatory impacts of SPDD.

To further characterize the features of DEGs, the Gene Ontology (GO) database provides a comprehensive description of gene and gene product attributes in biological systems, encompassing three major categories: Molecular Function (MF), Cellular Component (CC), and Biological Process (BP). Figure 4D illustrates representative GO functional sets for each category. The analysis revealed that, with the Model group serving as the reference genome, the molecular functions of differentially expressed genes in the experimental cohort were primarily associated with enzyme activity and ion channel activity. Additionally, the biological processes were mainly related to muscle system process, regulation of ion transmembrane transport, and signal release.

To further elucidate the functional characteristics of DEGs, KEGG functional annotation analysis was conducted on the DEGs between the Model group and the SPDD group. Figure 4E,F illustrate the 20 most significantly enriched pathways among the DEGs based on KEGG enrichment analysis, including the IL-17 signaling pathways (*p*-value = 0.00049) and MAPK signaling pathways (*p*-value = 0.00241), which are intricately associated with inflammation. Upon analysis of the DEGs within these enriched pathways, it was intriguing to observe that the IL-17 signaling pathway was enriched with inflammation-related genes such as MAPK4, CXCL1, and S100A9. Therefore, in this study, particular attention was focused on the IL-17 signaling pathway and the differentially expressed gene MAPK4 (Figure 4G).

### 3.5. SPDD Inhibited MAPK4/AKT Signaling in DSS-Induced Colitis

**Figure 5:**
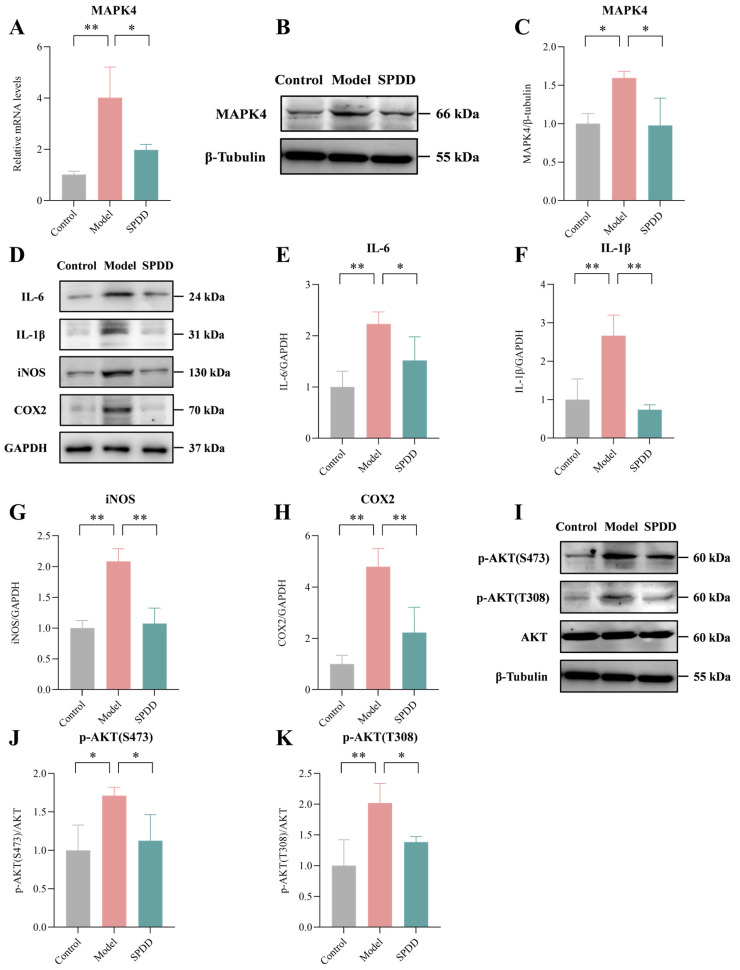
SPDD Inhibited MAPK4/AKT Signaling in DSS-Induced Colitis. (**A**) mRNA expression levels of MAPK4. (**B**) Protein expression levels. (**C**) Protein expression level of MAPK4. (**D**) The protein expression of inflammatory factors. (**E**) Protein expression level of IL-6, (**F**) IL-1β, (**G**) iNOS, and (**H**) COX-2. (**I**) Protein expression level. (**J**) Protein expression level of p-AKT(S473), and (**K**) p-AKT(T308). Values are mean ± SEM (*n* = 3). * *p* < 0.05 and ** *p* < 0.01 indicate comparisons between the Model group and the Control or SPDD group.

This study initially investigated the impact of SPDD on MAPK4, employing molecular docking analysis to ascertain whether MAPK4 is a potential target of SPDD. The docking results revealed a binding energy of −71.2353 kcal/mol between SPDD and MAPK4, indicating a high affinity and supporting the likelihood of MAPK4 serving as a target for SPDD (Figure S1, Table S3). Further analysis of MAPK4 mRNA expression levels demonstrated a significant upregulation in the model group compared to the control group (*p* < 0.01), which was markedly suppressed by SPDD intervention, suggesting that SPDD may regulate the expression of MAPK4 (*p* < 0.05) (Figure 5A). Concurrently, compared with the model group, the expression level of MAPK4 protein was also reduced by SPDD treatment (Figure 5B,C).

Furthermore, the protein expression levels of these factors were quantified. The study found that in the model group, the protein expression levels of *IL-6*, *IL-1β*, *iNOS*, and *COX-2* were significantly elevated compared to the control group (*p* < 0.01). Notably, SPDD intervention significantly attenuated the expression of these inflammatory mediators, indicative of its potential anti-inflammatory efficacy (*p* < 0.05) (Figure 5D–H).

Additionally, the results demonstrated a significant increase in AKT protein phosphorylation in the model group (*p* < 0.05). In contrast, the SPDD treatment group exhibited a marked suppression of AKT phosphorylation levels (*p* < 0.05) (Figure 5I–K). This finding suggests that SPDD potentially exerts its effects by inhibiting the activation of the MAPK4/AKT signaling pathway.

### 3.6. SPDD Inhibited the Interaction Between MAPK4 and AKT, Reducing the Phosphorylation Level of AKT

**Figure 6:**
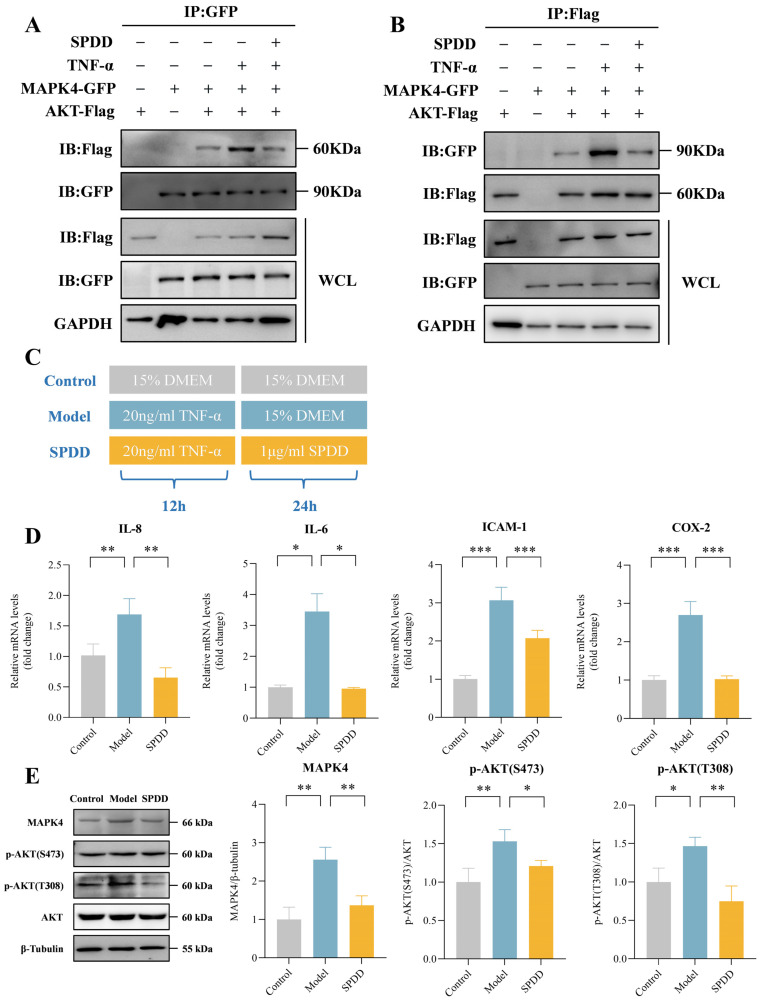

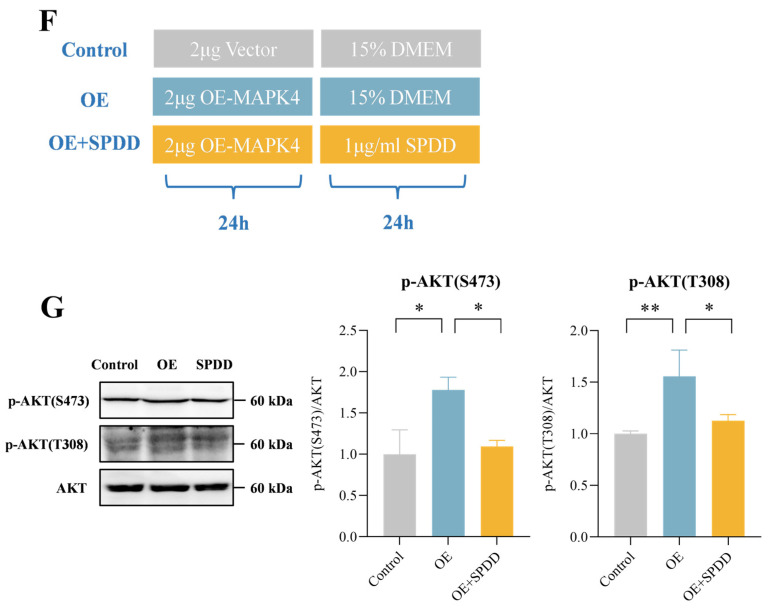
SPDD Inhibited the Interaction between MAPK4 and AKT, Reducing the Phosphorylation Level of AKT. (**A**,**B**) MAPK4 and AKT interactions. (**C**) Cellular experiment. (**D**) mRNA levels of IL-8, IL-6, ICAM-1, and COX-2 in Caco-2 cells under TNF-α treatment in the absence or presence of SPDD. (**E**) MAPK4, p-AKT(S473), and p-AKT(T308) expressions in Caco-2 cells under TNF-α were measured by immunoblots and quantified. (**F**) Overexpression experiment. (**G**) MAPK4 overexpression, p-AKT(S473), and p-AKT(T308) expressions in Caco-2 cells under TNF-α were measured by immunoblots and quantified. Values are mean ± SEM (*n* = 3). * *p* < 0.05; ** *p* < 0.01 and *** *p* < 0.001 indicate comparisons between the Model group and the Control or SPDD group.

To further investigate the interaction between MAPK4 and AKT, Co-IP technology was employed for an in-depth study. In the experiment, Caco-2 cells were co-transfected with the MAPK4-GFP and AKT-Flag plasmids. From the results, it is evident that TNF-α enhances the interaction between MAPK4 and AKT, while SPDD markedly attenuates this interaction. From the results, it is evident that TNF-α enhances the interaction between MAPK4 and AKT, while SPDD markedly attenuates this interaction (Figure 6A,B).

Furthermore, in cellular assays, the experimental design is shown in Figure 6C. TNF-α stimulation led to a significant increase in the mRNA expression of inflammatory markers, including *IL-8*, *IL-6*, *ICAM-1*, and *COX-2*. SPDD supplementation significantly reduced the levels of these inflammatory markers (*p* < 0.05) (Figure 6D).

Additionally, it was found that SPDD treatment was associated with a reduction in MAPK4 and the phosphorylation of AKT proteins in Caco-2 cells (Figure 6E). In MAPK4 overexpression experiments (Figure 6F), SPDD intervention led to a significant decrease in AKT protein phosphorylation levels (*p* < 0.05) (Figure 6G). These findings further confirm that SPDD exerts its anti-inflammatory effects by inhibiting the MAPK4/AKT signaling pathway. In summary, SPDD exhibits a potent inhibitory effect on TNF-α-induced inflammation in Caco-2 cells.

## 4. Discussion

UC is a gastrointestinal disorder characterized by an escalating global incidence, particularly pronounced in densely populated nations [41,42]. In the treatment of UC, 5-ASA drugs and corticosteroids are the primary therapeutic modalities for inducing remission; however, their remission rates typically do not exceed 20–30%. The key to surpassing this limitation lies in integrating therapeutic strategies with precise, personalized medication. Recent studies have indicated the potential significance of active components from natural products in the treatment of colitis. In this study, we have isolated and purified SPDD from rose bee pollen and found it to have a significant alleviating effect on colitis.

The target compound was separated and purified using preparative HPLC, which was further analyzed and identified using LC-Q-TOF MS. Through precise mass and fragmentation pattern analysis, we successfully identified the main compound in the fractions, and its structure was determined to be di-*p*-coumaroyl-caffeoyl spermidine. The purity of the compound exceeded 98%, confirming the effectiveness of our purification strategy in obtaining a high-purity bioactive compound. Spermidine has been thoroughly verified to possess various activities such as cardiovascular protection, neuroprotection, anti-tumor, and anti-inflammatory effects [43,44]. The focus of this article is on SPDD, which is formed by combining spermidine as the basic framework with phenolic acids. Numerous studies have demonstrated the significant protective effects of phenolic acids on colitis [45]. However, regarding the SPDD studied in this paper, there is almost no research on its protective effect on colitis and its mechanism of action. Based on this, we propose a bold hypothesis: SPDD may exhibit superior efficacy in alleviating colitis and warrants further exploration and investigation.

The DSS-induced UC model, exhibiting human-like symptoms and capable of disrupting the intestinal barrier, is used for preclinical drug testing [46,47]. Research indicates that the intestinal barrier is primarily composed of intestinal epithelial cells, including enterocytes, goblet cells, and Paneth cells, as well as tight junction (TJ) proteins, such as Occludin, ZO-1, and Claudin. Abnormal expression or dysfunction of TJ proteins and disruption of their integrity can lead to UC [48,49]. MUC2, a secretory mucin produced by goblet cells, is the primary macromolecular component of the intestinal mucus [50]. When the Muc2 mucus layer is damaged, pathogenic bacteria and inflammatory factors may penetrate the intestinal barrier, leading to colitis [51,52]. Our results showed that DSS treatment significantly decreased the expression of Occludin and ZO-1, while SPDD intervention reversed these changes. Moreover, SPDD significantly increased Muc2 expression, suggesting that SPDD aids in restoring goblet cell differentiation and protecting the intestinal mucosal barrier. In addition, SPDD exerts a significant inhibitory effect on the phenomenon of colon shortening induced by colitis and promotes the recovery of goblet cell numbers and the reconstruction of crypt architecture. These findings support the potential protective role of SPDD in maintaining and restoring intestinal barrier integrity in the UC mouse model.

However, the molecular mechanisms by which SPDD alleviates DSS-induced colitis in mice remain unclear. Through transcriptome sequencing analysis, a key atypical member of the MAPK family, MAPK4, has been identified, and its potential as a key target for SPDD’s function has been preliminarily confirmed through molecular docking experiments. The results indicate that SPDD and MAPK4 interact with protein residues (ARG, SER, GLY, LEU, ASN, GLU, PRO) through hydrogen bonds, van der Waals forces, carbon-hydrogen interactions, and amide-π stacking, thereby enhancing the stability of the complex. However, which domain of MAPK4 SPDD binds to, the binding sites shown by molecular docking, and which of these affect the activity and function of MAPK4 as active sites all require further research.

Emerging research underscores the significance of MAPK4 in the modulation of inflammatory responses. In diverse cancer types, such as triple-negative breast cancer [53], glioblastoma [54], and prostate cancer [55]. The research findings show that SPDD can significantly reduce the expression of MAPK4. Furthermore, by using TNF-α to establish an inflammatory model in Caco-2 cells, it has been further confirmed that SPDD can indeed alleviate colitis in mice by downregulating the expression of MAPK4. Our results further suggest that MAPK4 may serve as a candidate target for the treatment of UC.

In addition, previous studies have also shown that MAPK4 mainly functions as a kinase and regulates AKT through signal transduction cascades, reducing AKT phosphorylation [55]. The results of this study show that SPDD downregulates the expression of MAPK4. In addition, SPDD inhibits the interaction between MAPK4 and AKT, thereby reducing AKT phosphorylation. Given that SPDD can interact with MAPK4, we hypothesize that the binding of SPDD may affect the structure of MAPK4, leading to a weakened ability of MAPK4 to bind with AKT.

Research indicates that upon activation, AKT modulates the activity of NF-κB through a signaling cascade, which in turn leads to the expression of downstream inflammatory factors such as *IL-6*, *IL-1β*, *iNOS*, and *COX-2*, thereby playing a role in the inflammatory response [56,57,58]. Our study reveals that SPDD significantly affects the phosphorylation levels of AKT kinase, leading to a remarkable decline in the expression levels of inflammatory factors such as *IL-6*, *IL-1β*, *iNOS*, and *COX-2*. Meanwhile, utilizing a TNF-α-induced inflammation cell model in Caco-2 cells, it was demonstrated that SPDD effectively alleviates TNF-α-induced cellular inflammatory responses by inhibiting the MAPK4/AKT signaling pathway.

However, there are still many research limitations regarding the relationship between Spermidine (SPD) and SPDD. Firstly, it has been demonstrated that spermidine and phenolic acids (such as coumaric acid and caffeic acid) both possess anti-inflammatory properties [12,59]. The structural modification of spermidine derivatives may further enhance their interaction with inflammation-related targets, thereby improving their anti-inflammatory activity. Additionally, spermidine derivatives may exert their effects through more specific molecular mechanisms, reducing non-specific side effects and thus enhancing therapeutic efficacy. However, these hypotheses are still in the preliminary stage, and specific outcomes require further investigation. Furthermore, SPD, a widely distributed endogenous polyamine in living organisms, is synthesized via a two-step reaction from S-adenosylmethionine (SAM) and putrescine (PUT) and plays a crucial role in the regulation of cellular homeostasis [17]. Our study revealed that SPDD (with coumaric acid, caffeic acid, and SPD as precursors) significantly alleviated inflammatory responses in colitis mice, suggesting that exogenous supplementation of SPDD may compensate for the deficiency of endogenous SPD under disease conditions. However, due to the complexity of metabolism and current research limitations, it still needs to confirm the natural biosynthetic pathways of SPDD in the human body. Future research will focus on analyzing the differences in levels and anti-inflammatory activities between SPD and SPDD, as well as exploring the underlying mechanisms of action.

In conclusion, this study successfully isolated and purified a spermidine derivative from rose bee pollen for the first time, offering novel insights into the bioactive components of rose bee pollen. This achievement not only deepens the understanding of the functional components in rose bee pollen but also provides important scientific evidence for the development of dietary supplements and health products based on these constituents.

## Data Availability

The original contributions presented in the study are included in the article/Supplementary Material, further inquiries can be directed to the corresponding author.

## Supplementary Materials

The following supporting information can be downloaded at: https://www.mdpi.com/article/10.3390/foods14071110/s1, Figure S1: Molecular docking schematic and binding sites of SPDD with MAPK4; Table S1: Compound information in rose bee pollen in negative ion mode; Table S2: Identification results of the LC-Q-TOF MS total ion current (TIC) chromatogram; Table S3: SPDD and MAPK4 docking result; Table S4: Primers sequence used for RT-qPCR.

## Abbreviations

SPDD, Spermidine derivative; UC, Ulcerative colitis; DAI, disease activity index; DSS, dextran sulfate sodium; ZO-1, zonulae occludens-1; Muc2, mucin 2; MAPK4, mitogen-activated protein kinase4; AKT, Akt kinase; IL-6, Interleukin-6; IL-1β, Interleukin-1 beta; iNOS, inducible Nitric Oxide Synthase; COX-2, Cyclooxygenase-2.
