# Supplementary material for: A Spermidine Derivative Ameliorates Dextran Sulfate Sodium-Induced Colitis in Mice by Inhibiting the MAPK4/AKT Signaling Pathway
Source: Foods. 2025 Mar 23;14(7):1110. doi: 10.3390/foods14071110 (PMC11988437; doi:10.3390/foods14071110)
Supplement: Supplementary file 1 [file foods-14-01110-s001.zip › foods-3517198-supplementary.pdf]

Supplementary Data

A Spermidine Derivative Ameliorates Dextran Sulfate Sodium-Induced Colitis in Mice by Inhibiting the MAPK4/AKT Signaling Pathway

Yuxin Zhang [1], Zeyuan Deng [1,2], Hongyan Li [1,2] and Zeyin Jiang [1,*]

[1] State Key Laboratory of Food Science and Resources, Nanchang University, Nanchang 330047, China; zyx05101999@163.com (Y.Z.); dengzy@ncu.edu.cn (Z.D.); lihongyan@ncu.edu.cn (H.L.)

[2] International Institute of Food Innovation, Nanchang University, Nanchang 330051, China

* Correspondence: jiangzy20230915@163.com

TABLE AND FIGURE CAPTIONS

Figures

Figure S1. Molecular docking schematic and binding sites of SPDD with MAPK4.

Tables

Table S1. Compound information in rose bee pollen in negative ion mode.

Table S2. Identification results of the LC-Q-TOF MS total ion current (TIC) chromatogram.

Table S3. SPDD and MAPK4 docking result.

Table S4. Primers sequence used for RT-qPCR.

Figure S1.

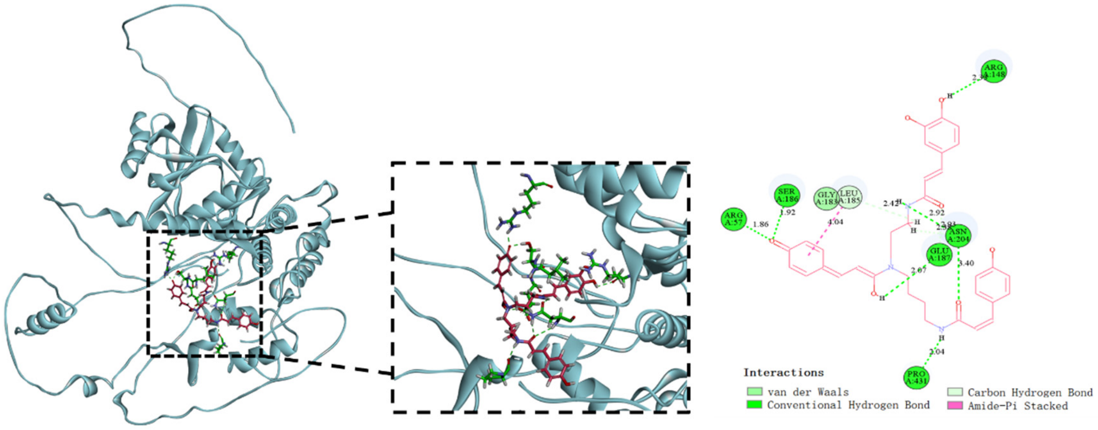
ARG A:148
SER A:186
GLY A:183
LEU A:185
ARG A:57
ASN A:204
GLU A:187
PRO A:431
1.86
1.92
4.04
2.42
2.92
3.40
2.07
2.04
Interactions
van der Waals
Conventional Hydrogen Bond
Carbon Hydrogen Bond
Amide-Pi Stacked

Table S1.

Peak	Formula	$[M-H]^-/(m/z)$	MS/MS/(m/z)	Identification
1	$C_{34}H_{37}N_3O_8$	614.2556	119.0516、316.1704、358.1454、478.2029	*p*-coumaroyl-di-caffeoyl spermidine
2	$C_{34}H_{37}N_3O_7$	598.2557	119.0503、316.1665、358.1412、478.1987	di-*p*-coumaroyl-caffeoyl spermidine
3	$C_{34}H_{37}N_3O_6$	582.2690	119.0526、316.1719、342.1515、462.2098	tri-*p*-coumaroyl spermidine

Table S2.

Peak	Formula	$[M-H]^-$ /(m/z)	Major MS/MS fragment ions(m/z)	Identification
1、2、3	$C_{34}H_{37}N_3O_7$	598.2573	119.0500、316.1660、358.1407、478.1982	di-*p*-coumaroyl-caffeoyl spermidine
4	$C_{34}H_{37}N_3O_6$	582.2613	119.0499、316.1656、342.1448、462.2028	tri-*p*-coumaroyl spermidine

Table S3.

Pose Number	1	2	3	4	5	6
-cdocker energy(kcal/mol)	48.8726	42.3587	45.0446	37.4807	18.9019	27.0022
-cdocker internation energy(kcal/mol)	71.2353	60.9260	59.7210	59.6672	48.9101	47.3648

Table S4.

NCBI ID	Primer name	Sequence (5'-3')
MAPK4-M	*Forward primer*	ACCTTGTGCTCAAGATTGG
	Reverse primer	CTTCTGACAGATAACCCTTGTG
β- actin-M	*Forward primer*	CTACCTCATGAAGATCCTGACC
	Reverse primer	CACAGCTTCTCTTTGATGTCAC
IL8-H	*Forward primer*	ACTGAGAGTGATTGAGAGTGGAC
	Reverse primer	AACCCTCTGCACCCAGTTTTC
IL6-H	*Forward primer*	AGCCACTCACCTCTTCAGAAC
	Reverse primer	ACATGTCTCCTTTCTCAGGGC
ICAM-1-H	*Forward primer*	GGAAATACTGAAACTTGCTGCCTAT
	Reverse primer	ACACATGTCTATGGAGGGCCAC
COX2-H	*Forward primer*	CTGGCGCTCAGCCATACAG
	Reverse primer	CGCACTTATACTGGTCAAATCCC
β- actin-H	*Forward primer*	CGTGCGTGACATTAAGGAGA
	Reverse primer	ATACTCCTGCTTGCTGATCCA
